# Angioplastias da Artéria Mamária Interna Esquerda e Direita em Paciente de 3 Anos com Doença de Kawasaki e Falha na Cirurgia de Revascularização do Miocárdio

**DOI:** 10.36660/abc.20190904

**Published:** 2020-05-22

**Authors:** René Hameau, Daniel Springmuller, Francisco Garay, Alberto Fuensalida, Gonzalo Martinez

**Affiliations:** Hospital Clinico Universidad Catolica Santiago Chile Hospital Clinico Universidad Catolica, Santiago - Chile

**Keywords:** Cardiopatias Congênitas/complicações, Doença de Kawasaki/complicações, Revascularização do Miocárdio/cirurgia, Aneurisma Cardíaco/cirurgia, Intervenção Coronária Percutânea/métodos, Terapia Trombolítica/métodos

**Figura 1:**
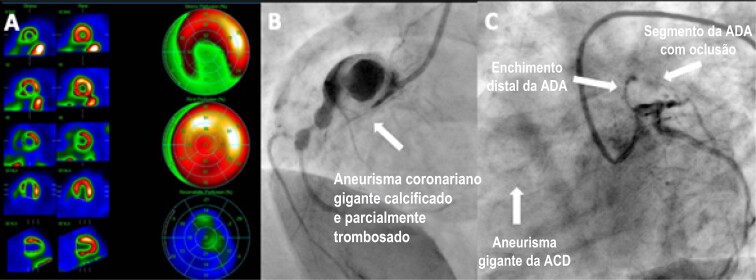
– *A) SPECT mostrando área do VE com 45% de isquemia; B) ACD; C) Artéria coronária esquerda.*

Um menino de 3 anos e 5 meses de idade com histórico de doença de Kawasaki diagnosticada aos 6 meses de idade e infarto do miocárdio sem supradesnivelamento do segmento ST (IAMSSST) foi submetido à cirurgia de revascularização do miocárdio (CRM) devido a um grande defeito de perfusão e disfunção sistólica moderada na Tomografia computadorizada por emissão de fóton único (SPECT) (Figura 1A) e uma angiocoronariografia mostrando um aneurisma gigante parcialmente trombosado na artéria coronária direita (ACD) e uma oclusão completa da artéria descendente anterior (ADA) proximal esquerda (Figuras 1B-C ) Foi realizado enxerto da artéria mamária interna esquerda (AMIE) à ADA e anastomose livre da artéria mamária interna direita (AMID) para a artéria descendente posterior (ADP) da ACD.

**Figura 2:**
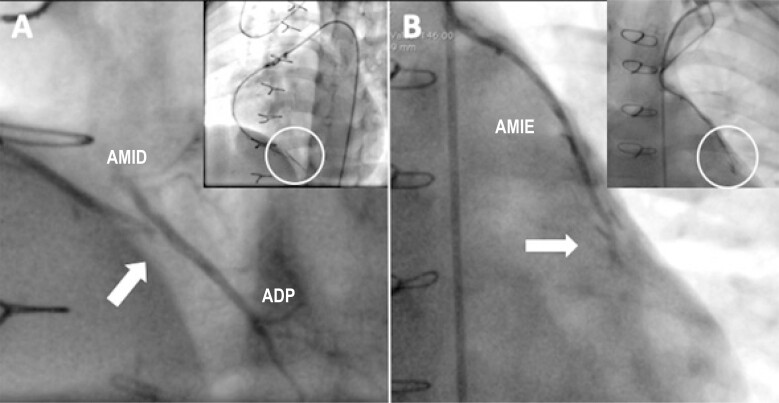
– *A) Estenose grave na anastomose da AMID distal à ADP; B) Oclusão total da AMIE.*

Três meses depois, o SPECT mostrou 16% de isquemia do ventrículo esquerdo (VE). A angiocoronariografia revelou lesão grave na anastomose distal da AMID para a ADP e oclusão completa na AMIE distal (Figura Suplementar S1). Decidiu-se por realizar a intervenção percutânea ad hoc. Um cateter guia 5F JR3.5 foi utilizado para o engate seletivo da AMID. Um fio Runthrough (Terumo Corporation, Tóquio, Japão) foi avançado e a angioplastia com balão simples (ABS) foi realizada com balões semi-complacentes de 1,25 x 12 e 1,5 x 15, obtendo um resultado favorável (Figura 2-A). Posteriormente, o guia JR3.5 foi posicionado na subclávia esquerda, a partir da qual o fio Runthrough foi avançado até a AMIE, seguido por um microcateter 1.8F Finecross (Terumo Corporation, Tóquio, Japão), que permitiu atravessar toda a oclusão. Posteriormente, devido à incapacidade de engate seletivo da AMIE, todas as injeções do meio de contraste foram realizadas através do micro-cateter.

**Figura 3:**
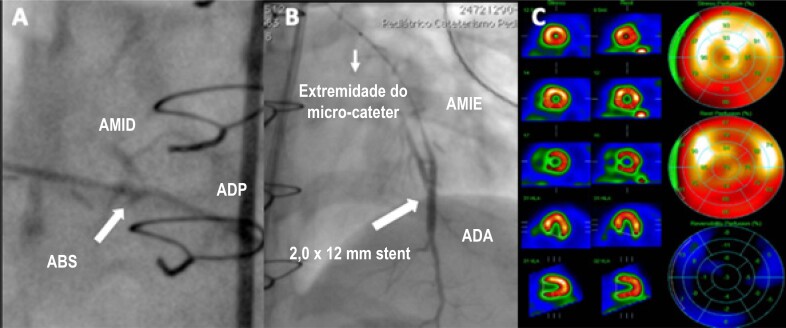
– *Resultados pós-procedimento. A) ABS para AMID-ADP; B) Stent para AMIE-ADA; C) SPECT de seguimento mostrando leve defeito de perfusão.*

Várias angioplastias com balão resultaram em estenose residual significativa, portanto, um stent eluidor de Zotarolimus de 2,0 x 12 foi implantado com sucesso (Figura 2-B). No seguimento de três meses, o SPECT não mostrou isquemia significativa, confirmando ótimos resultados pós-procedimento (Figura 3-C).

Os riscos de grandes eventos cardiovasculares adversos podem chegar a 48% em pacientes pediátricos com aneurismas coronarianos gigantes devido à doença de Kawasaki.^1^ A intervenção coronária percutânea (ICP), a revascularização miocárdica e trombólise sistêmica foram utilizadas,^2^ mas não há relatos de reintervenção após a revascularização miocárdica. A ICP nesse cenário é complexa devido a dificuldades técnicas, experiência limitada e necessidade de adaptar dispositivos adultos a crianças pequenas. A avaliação e a intervenção multidisciplinares (abrangendo especialistas em adultos e pediatria) são fundamentais para o sucesso dos procedimentos.
