# Supplementary material for: Angioplastias da Artéria Mamária Interna Esquerda e Direita em Paciente de 3 Anos com Doença de Kawasaki e Falha na Cirurgia de Revascularização do Miocárdio
Source: Arq Bras Cardiol. 2020 May 22;114(5):846–8. [Article in Portuguese] doi: 10.36660/abc.20190904 (PMC8386994; doi:10.36660/abc.20190904)
Supplement: Translation

# Translation: Left and Right Internal Mammary Artery Angioplasties in a 3-year-old Patient with Kawasaki Disease and Failed Coronary Artery Bypass Graft Surgery

**Authors:** René Hameau, Daniel Springmuller, Francisco Garay, Alberto Fuensalida, Gonzalo Martinez

**Affiliations:** Chile Hospital Clinico Universidad Catolica, Santiago - Chile

**Keywords:** Heart Defects, Congenital/complications, Kawasaki Disease/complications, Myocardial Revascularization/surgery, Heart Aneurysm/surgery, Percutaneous Coronary Intervention/methods, Thrombolytic Therapy/methods

A 3 years and 5 months old boy with a history of Kawasaki disease diagnosed at 6 months of age and a previous Non-ST myocardial infarction (NSTEMI) underwent Coronary Artery Bypass Graft (CABG) surgery due to a large perfusion defect and moderate systolic dysfunction on Single-photon emission computed tomography (SPECT) (Figure 1A) and a coronary angiogram showing a partially thrombosed giant aneurysm on the Right coronary artery (RCA) and a complete occlusion of the proximal Left anterior descending artery (LAD) (Figures 1B-C). A Left internal mammary artery (LIMA) graft to LAD and a free Right internal mammary artery (RIMA) to Posterior descending artery (PDA) anastomosis of RCA was performed.

**Figure 1:** – *A) SPECT showing 45% area of LV ischemia; B) RCA; C) Left coronary artery.*

Three months later, SPECT showed a 16% left ventricle (LV) ischemia. Coronary angiography revealed a severe lesion at the RIMA to PDA distal anastomosis and a complete occlusion at the distal LIMA (Supplemental Figure S1). Ad-hoc percutaneous intervention was decided. A 5F JR3.5 guiding catheter was used to selectively engage the RIMA. A Runthrough wire (Terumo Corporation, Tokyo, Japan) was advanced and Plain old balloon angioplasty (POBA) was performed with 1.25 x 12 and 1.5 x 15 semi-compliant balloons, achieving a favorable result (Figure 2-A). Subsequently, the JR3.5 guide was positioned in the left subclavian, from which the Runthrough wire was advanced into the LIMA, followed by a 1.8F Finecross microcatheter (Terumo Corporation, Tokyo, Japan), which allowed crossing the entire occlusion. Thereafter, due to the inability to selectively engage the LIMA, all contrast media injections were performed through the microcatheter.

**Figure 2:** – *A) Severe stenosis at the distal RIMA to PDA anastomosis; B) Total LIMA occlusion.*

Several balloon angioplasties have resulted in significant residual stenosis, thus a 2.0 x12 Zotarolimus-eluting stent was successfully deployed (Figure 2-B). At a 3-month follow-up, SPECT showed no significant ischemia, confirming optimal post-procedural results (Figure 3-C).

**Figure 3:** – *Post-procedural results. A) POBA to RIMA-PDA; B) Stenting to LIMA-LAD; C) Follow-up SPECT with mild perfusion defect.*

The risks of major adverse cardiovascular events can reach up to 48% in pediatric patients with giant coronary aneurysms due to Kawasaki disease.^1^ Percutaneous coronary intervention (PCI), CABG and systemic thrombolysis have been used,^2^ yet no reports of re-intervention after CABG are available. PCI in this setting is complex due to technical difficulties, limited experience and the need to adapt adult devices to small children. Multidisciplinary assessment and intervention (encompassing adult and pediatric specialists) are key for procedural success.
